# The Course of Anti-HBc Antibodies over Time in Immunocompromised Hosts

**DOI:** 10.3390/vaccines10020137

**Published:** 2022-01-18

**Authors:** Caroline Holtkamp, Melanie Fiedler, Ulf Dittmer, Olympia E. Anastasiou

**Affiliations:** Institute for Virology, Essen University Hospital, Medical Faculty, University of Duisburg-Essen, 45147 Essen, Germany; caroline.holtkamp@uk-essen.de (C.H.); melanie.fiedler@uk-essen.de (M.F.); ulf.dittmer@uk-essen.de (U.D.)

**Keywords:** anti-HBc, HBV, immunocompromised, immunosuppression, occult hepatitis B

## Abstract

Hepatitis B virus infection results in the appearance of anti-HBc antibodies that normally persist lifelong. We analyzed the course of anti-HBc antibodies overtime, focusing on patients with a permanent loss or fluctuating anti-HBc antibodies. From 120,531 patients tested for anti-HBc antibodies (Architect, Abbott) from January 2006 to December 2020, ≥4 serial values were available in 8098 and permanent or intermittent anti-HBc loss was observed in 139 patients. It was relatively frequent in baseline anti-HBc positive, immunocompromised patients with available serial measurements of anti-HBc overtime (13% of hematologic/oncologic patients, 10% of solid organ transplant recipients, and 6% of HIV patients compared to 3% in patients with other diseases). In the same period, 12,607 samples were tested for HBsAg, anti-HBc antibodies, and HBV DNA—in nine cases we detected HBV DNA with undetectable anti-HBc and HBsAg. In four out of nine cases contamination of the PCR during processing was the likeliest cause, in another four, no further data were available, while in one the HBV DNA was later followed by a temporary anti-HBc seroconversion. In conclusion, permanent or intermittent anti-HBc loss is more common in immunocompromised hosts than in patients with other underlying diseases. Furthermore, anti-HBc and HBsAg assays can be safely used to exclude an active HBV infection, even in immunocompromised hosts.

## 1. Introduction

Hepatitis B virus (HBV), a partially double-stranded deoxyribonucleic acid (DNA) virus, is the cause of hepatitis B, a disease linked to considerable morbidity and mortality. A third of the population worldwide has an active or immunological controlled infection [1,2]. Germany is a country with a low HBV prevalence and this, above all, is because of vaccination against HBV, which has been available since the 1980s. Especially for immunocompromised patients, vaccination is important [3]. Due to its significance as a health issue, fast, reliable, and cheap diagnostic tools are crucial for public health and, indeed, today, screening markers such as hepatitis B surface antigen (HBsAg) and antibodies against the HBV core protein (anti-HBc) are readily at our disposal. Contact with the virus can be shown by the presence of anti-HBc antibodies, while the presence of HBsAg indicates an active acute or chronic infection [4]. Anti-HBc levels may vary at different phases of HBV infection, and tend to be significantly lower in patients with an immunologically controlled infection than in patients with an active infection [5]. Interestingly, there is also a correlation between the amount of anti-HBc antibodies and the measured HBV DNA level [6,7]. 

Due to the persistence of covalently closed circular DNA (cccDNA) in the liver, a reactivation of HBV infection is possible or even probable in immunosuppressed patients [8]. Anti-HBc antibodies, together with HBsAg, play an important role in identifying patients with a need for a more intensive follow-up or a prophylaxis against HBV reactivation [9], especially given the fact that anti-HBc antibodies are considered to persist lifelong [10]. Although the usefulness and dependability of anti-HBc antibodies as a marker for previous or ongoing HBV infection has been well documented, cases of HBV reactivation lacking detectable anti-HBc antibodies have been described [11,12]. Furthermore, a few cases with HBV DNA in the serum and simultaneously the absence of anti-HBc antibodies have been described in blood donors [13]. 

The aim of our study was to analyze the course of anti-HBc antibodies overtime in both HBsAg negative and positive patients, with a focus on patients with a permanent loss of anti-HBc antibodies or fluctuating anti-HBc antibodies.

## 2. Materials and Methods

**Figure 1:**
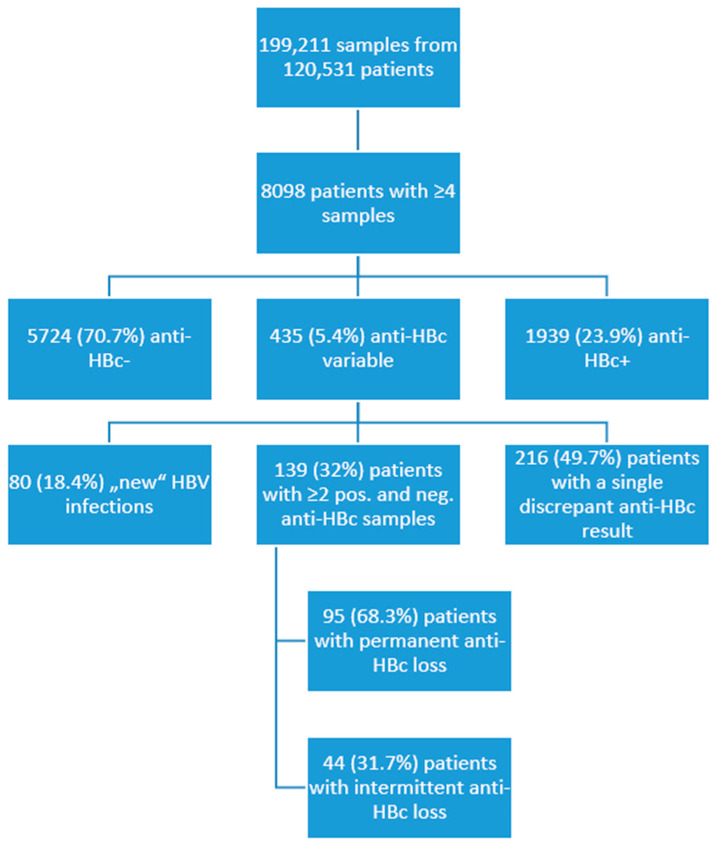
Flowchart of the patient cohort for 2006 to 2020.

Retrospectively, we evaluated the results of 199,211 samples from 120,531 patients, which were tested for anti-HBc antibodies as routine clinical diagnostic at the Institute for Virology, University Hospital Essen, from January 2006 to December 2020. There was no universally applied algorithm for patient screening, both due to the large timeframe and also the fact that the samples were collected from different departments. We looked for patients with serial measurements of anti-HBc antibodies and selected patients (*n* = 8098) with four or more measurements for further analysis. The majority were anti-HBc negative (*n* = 5724, 70.7%), some had consistently detectable anti-HBc antibodies (*n* = 1939, 23.9%), while in 435 (5.4%) patients, the anti-HBc antibodies varied (Figure 1). Variance of anti-HBc antibodies could be seen in cases of new HBV infections (*n* = 80, 18.4%), meaning a new detectable and then consistent anti-HBc, or as a single discrepant anti-HBc result in 216 (49.7%) patients. A group of 139 (32%) patients with at least two positive and two negative anti-HBc measurements, as well as detectable anti-HBc at baseline, was selected for further analysis. This criterion was chosen to minimize the risk that the discrepant anti-HBc measurement was due to technical (unspecific signal) or logistical (sample from another patient) errors. In addition, samples with implausible anti-HBc results and a S/CO value near a cut-off of 1 were centrifuged and measured again to minimize the cases of false positives. Values corresponding to measurements performed 13 to 15 years after baseline were grouped together due to the scarcity of results. The study was conducted in accordance with the ethical guidelines of the 1975 Helsinki Declaration and was approved by the ethics and research committees of the University Hospital Essen (15-6495-BO, 13 October 2015).

Samples were centrifuged and stored at 2–8 °C until measurement. Serological parameters (where available), such as HBsAg, anti-HBs, anti-HBe, HBeAg, and anti-HBc (IgM and IgG), were measured by chemiluminescence immunoassays using Architect (Abbott, Germany), according to the manufacturer’s instructions. For anti-HBc antibodies, a sample/cutoff (S/CO) <1.0 was considered to be negative and >1.0 was positive. 

HBV viral load (where available) was quantified by real-time PCR (real-time Abbott and Siemens bDNA HBV test platform) according to the manufacturer’s instructions. In thirty samples of 19 patients, anti-HBc antibodies were additionally measured using the Liaison XL assay (Diasorin, Saluggia, Italy). Continuous data were expressed as median (interquartile range (IQR)). Categorical data were described as frequencies of the subjects with a specific characteristic. Statistical significance was assessed by Chi-Square/Fisher’s Exact Test, Mann−Whitney test, Wilcoxon Signed Ranks test, and logistic regression, which were performed, as applicable, using SPSS (v27.0, SPSS Inc., Chicago, IL, USA). Two-tailed *p*-values less than 0.05 were considered statistically significant.

## 3. Results

### 3.1. The Anti-HBc S/CO Values Are Higher in HBsAg Positive vs. HBsAg Negative Patients and Remain Stable with a Slightly Declining Tendency Overtime

**Figure 2:**
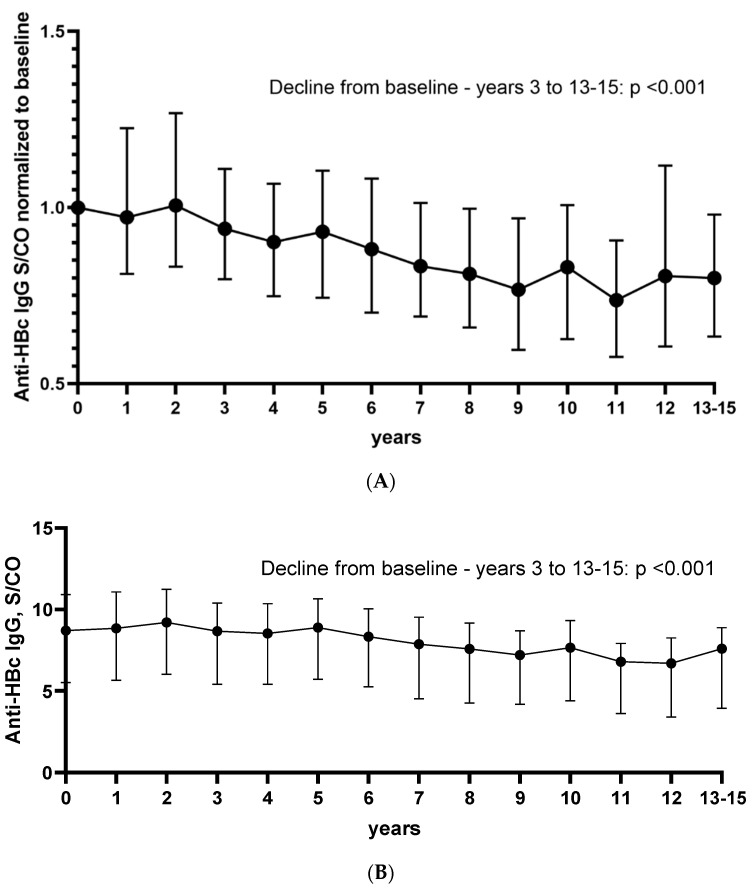

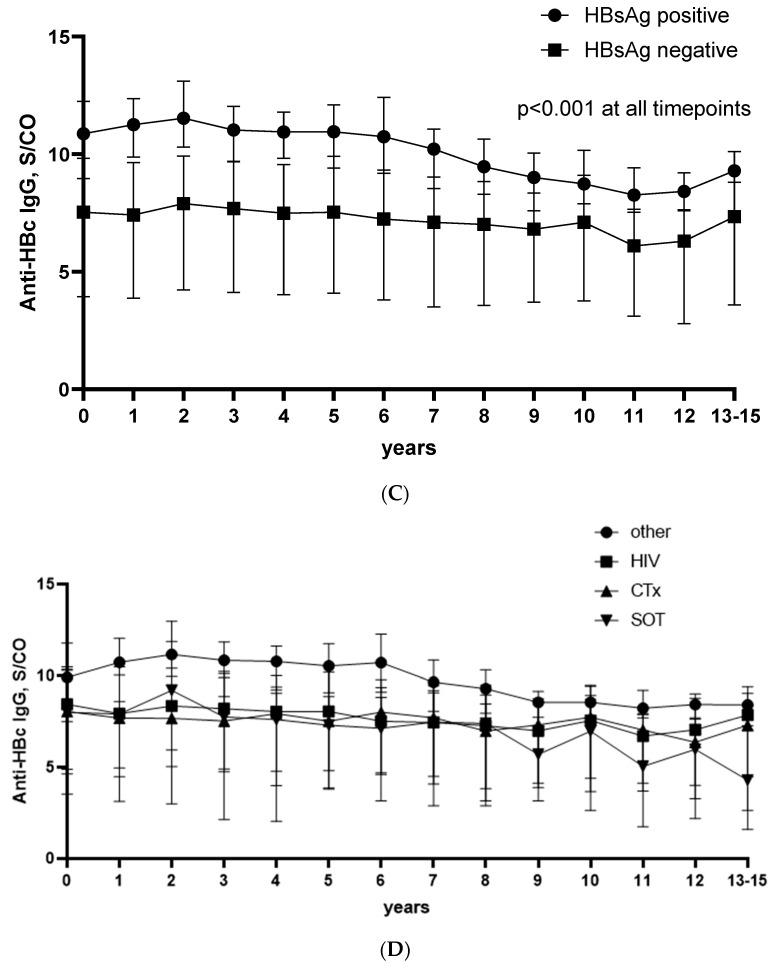
Anti-HBc S/CO values overtime either normalized to baseline (**A**) or in S/CO values (**B**) in all anti-HBc positive patients. Anti-HBc S/CO values overtime after stratification according to HBsAg status (**C**). Anti-HBc S/CO values overtime in patients with different underlying conditions (**D**). SOT—solid organ transplantation; CTx—patients with hematological or oncological malignancies after chemotherapy; HIV—human immunodeficiency virus.

Serial (≥4) anti-HBc antibody measurements were available in 8098 patients, of them 2130 had detectable anti-HBc antibodies at baseline and were thus further analyzed. The median S/CO values for each year are shown in Supplement (Table S1). The S/CO values were normalized to baseline and are presented in Figure 2A, while the anti-HBc S/CO values overtime are shown in Figure 2B. Baseline was compared to all other values. The anti-HBc S/CO values showed a slightly declining tendency over time. The decline was statistically significant for most comparisons (baseline vs. years 3, 4, 5, 6, 7, 8, 9, 10, 11, 12, and 13 to 15) to baseline (*p* < 0.001); however, the raw numerical difference was small (Figure 2B). In total, 71% of the patients had lower anti-HBc S/CO values at the last available measurement compared to baseline. In more detail, 53%, 49%, 62%, 66%, 63%, 67%, 73%, 75%, 76%, 75%, 80%, 64%, and 78% of patients had lower anti-HBc S/CO values at years 1, 2, 3, 4, 5, 6, 7, 8, 9, 10, 11, 12, and 13 to 15 compared to baseline. 

When comparing patients that were consistently HBsAg positive vs. negative, we observed that HBsAg positive patients had consistently higher anti-HBc S/CO values (*p* < 0.001) (Figure 2C). The statistical significance was preserved even after applying a Bonferroni correction for multiple testing. When comparing the anti-HBc levels in the different diseases, we detected lower anti-HBc levels in patients with immunosuppression, including HIV infected patients, solid organ transplant recipients, or hematologic/oncologic patients, than in patients with miscellaneous non-immunocompromising conditions (Figure 2D). 

### 3.2. Overall Anti-HBc Loss Is More Common in Immunocompromised Hosts Than in Patients with Other Underlying Diseases

Serial (≥4) anti-HBc antibody measurements were available in 8098 patients, and in 139 patients we had at least two positive and two negative anti-HBc measurements after excluding patients with an acute HBV infection (Figure 1). In about two thirds of the patients, the anti-HBc loss was permanent, while one third displayed only an intermittent anti-HBc loss. Of the 139 patients, 117 were immunosuppressed to some degree, with 51 (36.7%) having hematological or oncological malignancies and undergoing chemotherapy, 43 (30.9%) having HIV, 23 (16.5%) being solid organ transplantation recipients, and 22 (15.8%) had other underlying diseases and were considered immunocompetent, albeit not healthy, as the group included patients with renal, liver, or heart failure. 

**Figure 3:**
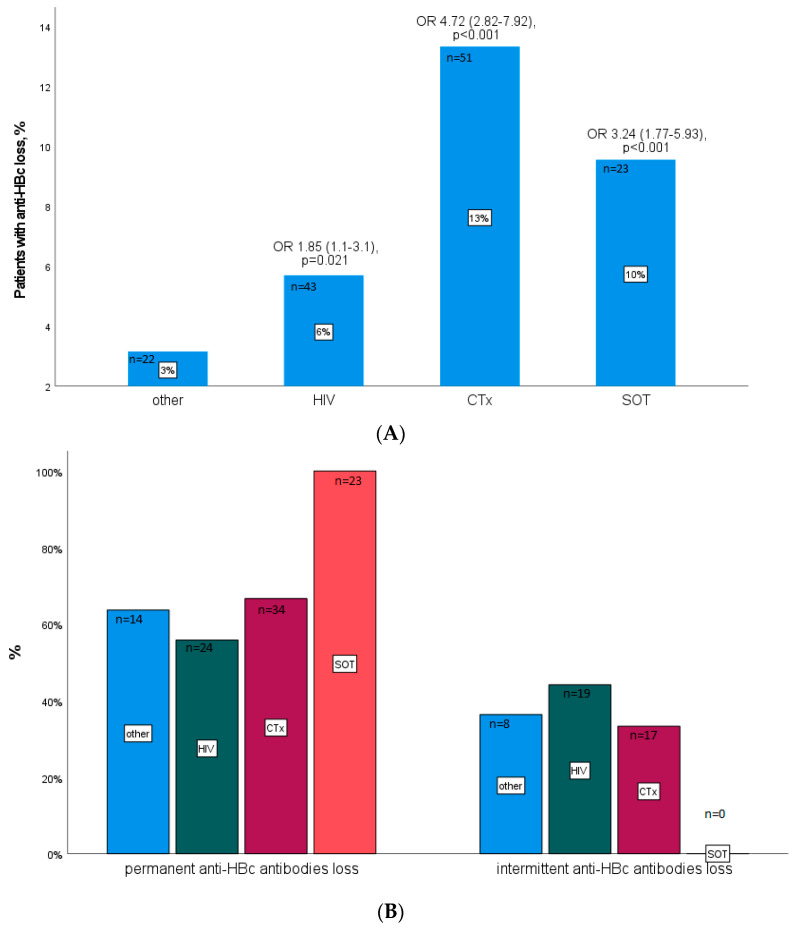
(**A**) Percentage of anti-HBc positive patients with anti-HBc loss stratified according to their underlying disease. (**B**) Percentage of patients with permanent vs. intermittent anti-HBc loss stratified according to their underlying disease. SOT—solid organ transplantation; CTx—patients with hematological or oncological malignancies after transplantation; OR—odds ratio; the 95% confidence interval is given in brackets.

Anti-HBc loss was significantly more common in immunosuppressed individuals. Evaluation of anti-HBc positive patients indicated that permanent or intermittent anti-HBc loss overtime could be observed in 13%, 10%, and 6% of hematological/oncological patients, solid organ transplant recipients, or HIV patients, respectively, in contrast to only 3% in patients with other underlying diseases (Figure 3A). A permanent anti-HBc loss was, in all, more frequently observed than an intermittent one, with comparable ratios for all groups except for the solid organ transplant recipients, in which the loss was always permanent (Figure 3B).

### 3.3. Results Are Largely Consistent in Different Test Systems 

In 30 samples taken from 19 patients (11 with permanent and 8 with intermittent anti-HBc loss), the anti-HBc antibodies were measured with two assay systems—Abbott and DiaSorin. The results were mostly consistent: 21 of 22 anti-HBc samples were negative in both assays, and 6 of 8 were positive in both assays. One sample was positive in the DiaSorin assay and negative in the Abbott assay, while the opposite was observed in two samples.

### 3.4. Anti-HBc and HBsAg Assays Can Be Safely Used to Exclude an Active HBV Infection

**Figure 4:**
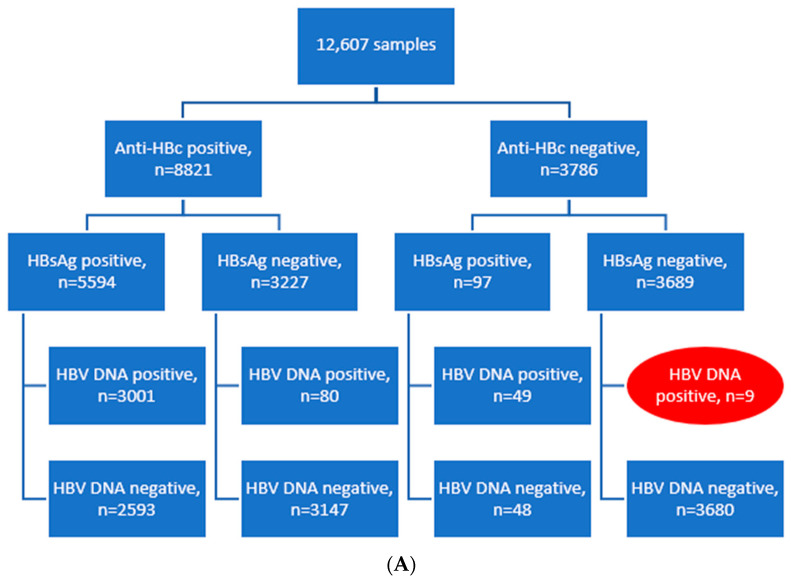

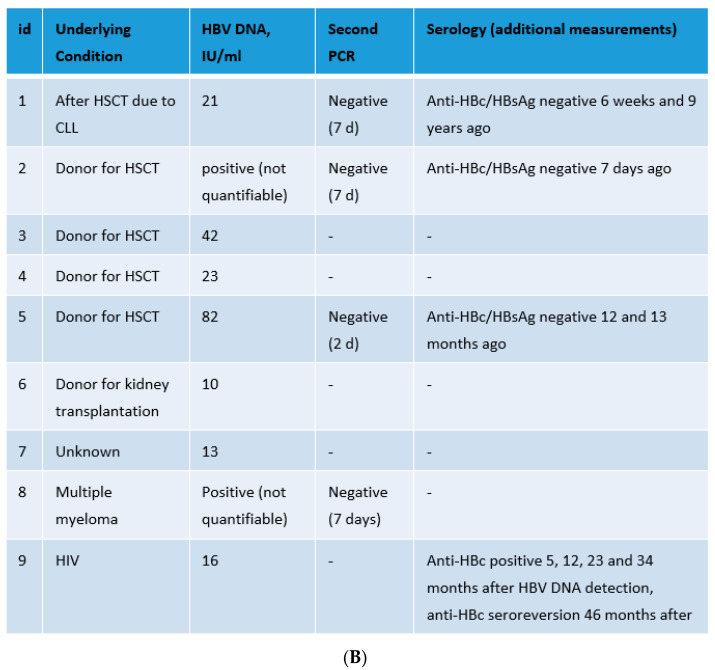
Panel (**A**): Evaluation of HBV serology and viral load in 12,607 samples. Panel (**B**): 9 patients being both anti-HBc and HBsAg negative but HBV DNA positive with underlying conditions and additional measurements. HSCT—hematopoietic stem-cell transplantation; CLL—chronic lymphocytic leukemia; HIV—human immunodeficiency virus.

HBsAg, anti-HBc, and HBV DNA were measured in 12,607 samples as a routine clinical diagnostic at the Institute for Virology, University Hospital Essen. Criterion for selection was the availability of all three of the above-mentioned parameters for the same sample. Occult hepatitis B (anti-HBc and HBV DNA positive, and HBsAg negative) was rare, as seen in Figure 4A, while anti-HBc negative HBV infection was even rarer, with 49 samples being HBsAg and HBV DNA positive and 9 being both anti-HBc and HBsAg negative but HBV DNA positive. Focusing on these nine cases, we observed that in all cases, the viral load was very low, and in four cases a second PCR was negative, making contamination the most plausible cause of HBV DNA detection. Only in one patient (id 9) was the HBV DNA detection probably correct, as this was followed by a temporary anti-HBc seroconversion (Figure 4B). Anti-HBc levels over time in the patient with id 9 are shown in Supplement (Figure S1).

## 4. Discussion

In this study, we characterized the clinical background of patients with a permanent or intermittent loss of anti-HBc antibodies. Anti-HBc antibodies are reported to persist lifelong [10]. Still, failure to detect anti-HBc antibodies in HBV infected immunocompromised hosts has been previously reported by both ours and by other groups [11,14]. In this study, permanent or intermittent loss of anti-HBc antibodies was more common in immunocompromised hosts than in patients with other underlying diseases—in 13% of hematologic or oncologic patients, 10% of patients after solid organ transplantation, and 6% of HIV infected patients. Loss of anti-HBc dropped to 3% in patients with other underlying illnesses. In total, permanent loss was seen more often than intermittent loss. Interestingly, all patients after solid organ transplantation had a permanent loss of anti-HBc antibodies. Anti-HBc loss has also been described previously by Ren et al. [15] in HBsAg negative patients after an average of 6 months after allo-HSCT. In contrast, the pattern of intermittent anti-HBc loss was frequently observed in HIV infected patients. In general, anti-HBc levels were lower in immunosuppressed patients than in patients without immunocompromising conditions.

Anti-HBc S/CO values were higher in HBsAg positive patients compared to HBsAg negative patients, and remained stable with a slight declining tendency overtime. This is in line with a previous study by Jia et al. [5], which interestingly included only immunocompetent patients. 

Our study pointed out that the anti-HBc results are largely consistent in different test systems, as we measured anti-HBc antibodies in some samples with different assays. The results are in line with a previous study, comparing anti-HBc assays for HBV screening and showing a good specificity and sensitivity of different anti-HBc assays for screening [16]. 

Testing for anti-HBc antibodies in all blood donors is actually established in many countries so as to exclude donors with an occult HBV infection [17,18]. Nevertheless, false positive [19,20] and false negative [11,13] anti-HBc results are described. False positive measurements in blood donors are challenging due to the deferral of these donors [19]. In contrast, in a few cases, blood donors were negative for HBsAg and anti-HBc antibodies, however HBV DNA was detected in the serum [13]. In our study, we detected 9 patients out of 12,607 tested samples that were at a time point both anti-HBc and HBsAg negative, but HBV DNA positive. In all cases, the viral load was very low. In 4 cases, a second PCR was negative, making contamination during processing the likeliest explanation for the unexpected result. Contamination due to user or device errors remains an issue, as reported before [21], and thus it should always be considered in case of discrepant results. However, in one patient (id 9), a case can be made for a true positive measurement of HBV DNA in the face of anti-HBc and HBsAg undetectability, as since this was followed by a temporary anti-HBc seroconversion (Figure 4B). In this patient, the initial diagnosis of the HIV infection was in 2016, simultaneously with the initial diagnosis of the HBV infection. Tenofovir therapy was started in April 2016, as recommended for HIV and HBV coinfected patients [22,23]. Unfortunately, additional sera were not available for verification of the results.

There are some limitations of our study. Due to its retrospective character, not all serological markers and HBV PCR were available for all of the samples. Furthermore, the anti-HBc antibodies were measured with a qualitative assay and, as such, we relied on a unstandardized, semiquantative, but a more readily available parameter, the S/CO value, to describe the overtime course of anti-HBc antibodies in different patient groups. The quantitative measurement of anti-HBc, especially in immunosuppressed patients, overtime would be interesting, and should be addressed in future prospective studies. Nevertheless, our study focuses on clinically significant issues and includes a great number of samples, including many derived from immunocompromised individuals, in which a reliable HBV diagnostic is critical. 

## 5. Conclusions

In conclusion, S/CO values of anti-HBc antibodies remain stable with a slight declining tendency overtime. Anti-HBc antibodies tend to be higher in HBsAg positive vs. negative individuals, and in immunocompetent vs. immunosuppressed ones. Permanent or intermittent anti-HBc loss is more common in immunocompromised hosts, with SOT recipients showing a permanent anti-HBc loss pattern, than in patients with other underlying diseases. Thus, a single anti-HBc antibody measurement may not be adequate to safely exclude an immunologically controlled, but currently not active HBV infection in immunocompromised hosts. The combination of anti-HBc antibodies and HBsAg, on the other hand, can be used to reliably exclude an active HBV infection, even in immunocompromised hosts.

## Data Availability

The data presented in this study are available in part upon request from the corresponding author. The data are not publicly available due to data protection issues.

## Supplementary Materials

The following supporting information can be downloaded at: https://www.mdpi.com/article/10.3390/vaccines10020137/s1, Figure S1: Anti-HBc (S/CO) levels in the patient with id 9 over time, Table S1: Anti-HBc S/CO values overtime.
