# Supplementary material for: The Course of Anti-HBc Antibodies over Time in Immunocompromised Hosts
Source: Vaccines (Basel). 2022 Jan 18;10(2):137. doi: 10.3390/vaccines10020137 (PMC8877063; doi:10.3390/vaccines10020137)
Supplement: Supplementary file 1 [file vaccines-10-00137-s001.zip › vaccines-1540887-supplementary.pdf]

vaccines

MDPI

Supplement:

Table S1: Anti-HBc S/CO values overtime.

	All	HBsAg positive	HBsAg negative
**Baseline**	8.71 (5.52-10.92)	10.87 (8.96-12.24)	7.53 (3.94-9.82)
**Year 1**	8.85 (5.65-11.085)	11.25 (9.88-12.36)	7.41 (3.87-9.64)
**Year 2**	9.21 (6.02-11.24)	11.53 (10.3-13.11)	7.9 (4.23-9.92)
**Year 3**	8.67 (5.4-10.39)	11.03 (9.68-12.03)	7.69 (4.12-9.69)
**Year 4**	8.53 (5.405-10.36)	10.94 (9.82-11.79)	7.49 (4.02-9.56)
**Year 5**	8.89 (5.715-10.65)	10.95 (9.41-12.09)	7.53 (4.08-9.9)
**Year 6**	8.335 (5.25-10.04)	10.74 (9.18-12.41)	7.24 (3.8-9.32)
**Year 7**	7.87 (4.55-9.525)	10.21 (8.54-11.06)	7.1 (3.5-9.03)
**Year 8**	7.575 (4.28-9.165)	9.47 (8.3-10.64)	7.01 (3.56-8.84)
**Year 9**	7.2 (4.21-8.7)	9.01 (7.6-10.05)	6.81 (3.71-8.34)
**Year 10**	7.65 (4.425-9.315)	8.74 (7.89-10.16)	7.1 (3.75-9.1)
**Year 11**	6.79 (3.63-7.92)	8.26 (7.53-9.42)	6.1 (3.11-7.66)
**Year 12**	6.695 (3.42-8.26)	8.42 (7.6-9.2)	6.3 (2.8-7.63)
**Year 13**	7.59 (3.955-8.875)	9.29 (7.59-10.11)	7.34 (3.59-8.8)

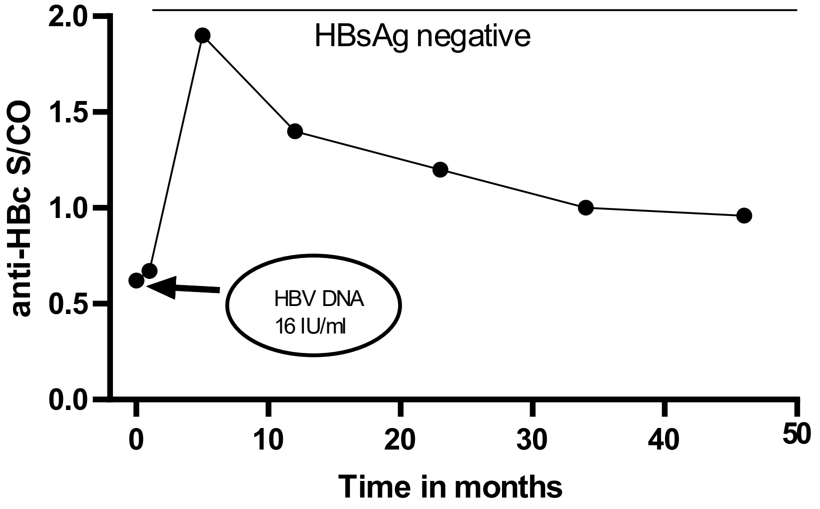

Figure S1: Anti-HBc (S/CO) levels in the patient with id 9 over time.
